# Irrelevant Features of a Stimulus Can Either Facilitate or Disrupt Performance in a Working Memory Task: The Role of Fluid Intelligence

**DOI:** 10.1371/journal.pone.0026249

**Published:** 2011-10-14

**Authors:** Bernardo Perfetti, Marcello Tesse, Sara Varanese, Aristide Saggino, Marco Onofrj

**Affiliations:** 1 Department of Physiology and Pharmacology, Sophie Davis School of Medicine, City College of New York, New York, New York, United States of America; 2 Department of Neuroscience and Imaging, University of Chieti-Pescara “G. d'Annunzio,” Chieti, Italy; 3 Division of Movement Disorders, Department of Neurology, New York University School of Medicine, New York, New York, United States of America; University of Regensburg, Germany

## Abstract

It has been shown that fluid intelligence (gf) is fundamental to overcome interference due to information of a previously encoded item along a task-*relevant* domain. However, the biasing effect of task-*irrelevant* dimensions is still unclear as well as its relation with gf. The present study aimed at clarifying these issues. Gf was assessed in 60 healthy subjects. In a different session, the same subjects performed two versions (letter-detection and spatial) of a three-back working memory task with a set of physically identical stimuli (letters) presented at different locations on the screen. In the letter-detection task, volunteers were asked to match stimuli on the basis of their identity whereas, in the spatial task, they were required to match items on their locations. Cross-domain bias was manipulated by pseudorandomly inserting a match between the current and the three back items on the irrelevant domain. Our findings showed that a task-irrelevant feature of a salient stimulus can actually bias the ongoing performance. We revealed that, at trials in which the current and the three-back items matched on the irrelevant domain, group accuracy was lower (interference). On the other hand, at trials in which the two items matched on both the relevant and irrelevant domains, the group showed an enhancement of the performance (facilitation). Furthermore, we demonstrated that individual differences in fluid intelligence covaries with the ability to override cross-domain interference in that higher gf subjects showed better performance at interference trials than low gf subjects. Altogether, our findings suggest that stimulus features irrelevant to the task can affect cognitive performance along the relevant domain and that gf plays an important role in protecting relevant memory contents from the hampering effect of such a bias.

## Introduction

In its psychometric definition Fluid intelligence, or gf, is considered a higher factor belonging to the second broad stratum of human cognitive abilities [1]. In the last decades a growing number of studies characterized the cognitive and neurobiological components of gf, leading to the idea that fluid intelligence is fundamental for the implementation of goal-directed behavior and for the optimization of individual performance [2]–[5]. The mechanisms through which this is achieved likely involve the construction of internal models in which representations of goals and information relevant to the task are actively maintained [6] and manipulated [7] in accordance to test instructions [2], [8], [9].

In light of the hypothesized function of gf, the ability to override interference due to irrelevant information acquires a particular importance. Indeed, susceptibility to interference has been investigated with several tasks in the context of Working Memory (WM). The main finding is that higher gf levels are associated with higher resistance to interference, suggesting that fluid intelligence is crucial for the shielding of task-relevant contents against the hampering effects of irrelevant information.

Despite the number of studies performed on this topic, only a specific form of interference has been investigated so far – i.e. the one exerted by task-relevant features of a previously encoded item – within the context of n-back tasks. On the other hand, the effects of task-irrelevant features of a stimulus on the ongoing process are poorly understood. The n-back is a recognition task in which subjects are asked to decide whether the item currently presented matches the stimulus seen *n* positions before [10], [11]. This is achieved by holding in memory a set of *n* serially ordered items that has to be updated every time a new stimulus is presented. Items must be matched on the basis of a specific characteristic, such as identity, screen location or color. In turn, each of these characteristics will constitute the *relevant* feature for the current task. When the presented stimulus does not match the *n*-back item, but matches a previously encoded stimulus presented *n* – 1 or *n* + 1 position before, the trial is called “lure”. In a three-back letter-identity task, an example of such trials is given by the following: N – ***H*** – J – H. When exposed to lure trials, subjects are usually less accurate and respond slower as a consequence of interference resolution [12]–[15]. In fact, the effect of familiarity might override the actual recollection of the stimulus leading to an incorrect response [13], [14], [16]. The example described above is a typical paradigm used to investigate interference control in WM. However, this kind of paradigm has an important limitation in that interference effects are manipulated and measured along a task relevant domain.

In the experiment at hand we intended to extend the previous work by examining whether task-*irrelevant* features of a previously processed item might bias the ongoing processing. Indeed, we ascertained whether the variation of the stimuli along an irrelevant dimension could influence the processing along the relevant domain. For instance, one can ask subjects to match items on their identity (*relevant domain or feature*) while having some stimuli presented *n* position before appearing at the same screen location (*irrelevant domain or feature*) of the current item. The question is: can the former bias the processing of the latter item? In other words, can an irrelevant characteristic of a salient item interfere with task execution? It is worth noting that, in this example, the location of the letter has little or no impact on task accomplishment in that, for efficiency in performance, subjects need to hold, maintain and process only the representations of stimuli identity. In addition to that, in cognitive psychology, spatial and verbal working memories are considered to be as two partially independent systems [17] (but [18]) and neuroimaging and lesions data have revealed that they share little neural resources ([19]–[21]; for a review of lesion studies: [22]). However, recent investigations have challenged this hypothesis showing that specific areas of the brain make domain-independent contribution to WM functioning [23], [24].

The investigation of cross-domain interference can provide important insights on the often neglect, reciprocal bias between domain specific WM components [25]. Furthermore, studying its relation with fluid intelligence will help the characterization of the cognitive mechanisms involved with the protection of task-relevant memory representations and the implementation of goal directed behavior.

The current study was designed to study this type of interference and to address the above-mentioned questions by ascertaining whether an *irrelevant* feature of a *salient* stimulus can disrupt or facilitate the processing of the current item. Furthermore, based upon the idea that gf promotes efficiency by biasing memory representations of task-relevant information [9] we expect that, if present, interference effects might be modulated by individual levels in fluid intelligence. To this end, we used two different versions (spatial and letter detection) of a three-back WM task with letters presented at different locations of the screen.

## Materials and Methods

### Participants

Sixty healthy normal subjects were recruited from the University of Chieti-Pescara. They ranged in age from 20 to 36 (mean ± SD, 23.7, ± 3) and their mean educational level was 13.6 (± 1.2). The sample consisted of 31 males and 29 females. All subjects gave written informed consent to participate in the study according to procedures approved by the local ethics committee of the University of Chieti-Pescara “G. d'Annunzio”. All participants were right handed, had normal or corrected to normal vision and none of them reported any history of psychiatric or neurological disorders.

### Working Memory Task

In the current study we administered the three-back working memory task [10], in which subjects viewed a continuous sequence of stimuli and have to decide whether each presented stimulus matches the item shown three stimuli earlier in the sequence. Subjects were instructed to respond with their right dominant hand by pressing one button for the “target” (“Yes” response), and another button for the “non-target” (“No” response) trials.

Subjects performed a letter-detection and a spatial version of the task. In order to avoid any possible confounds, in both conditions we adopted physically identical items (letters) shown at different locations of the screen. The task domain (letter-detection or spatial) was simply manipulated through the initial task instructions. In the letter-detection condition, we asked the subjects to retain and match the stimuli based upon the identity (letter) criteria while, in the spatial task, they were instructed to encode and match the stimuli by their positions. Notably, participants were explicitly told to ignore the task-irrelevant feature of the stimuli. This was done in order to suggest a strategy during task execution that might favor the active focusing on the task-relevant domain and enhancement of its representation in memory.

In order to measure cross-domain biasing effects, we manipulated some of the non-target trials (“No” responses). Specifically, on some non-target trials, the item presented on the screen matched the item seen three trials before on the irrelevant domain. For instance, in the letter-detection condition, some of the displayed letters matched the three-back item by its spatial location (irrelevant domain) but not by its identity. Similarly, in the spatial condition, stimuli on some trials did match the three-back stimulus by its identity (irrelevant domain) but not by its location. We labeled these kinds of non-target trials as “non-target lures”. All the others non-target trials were identified as “non-target control”.

**Figure 1:**
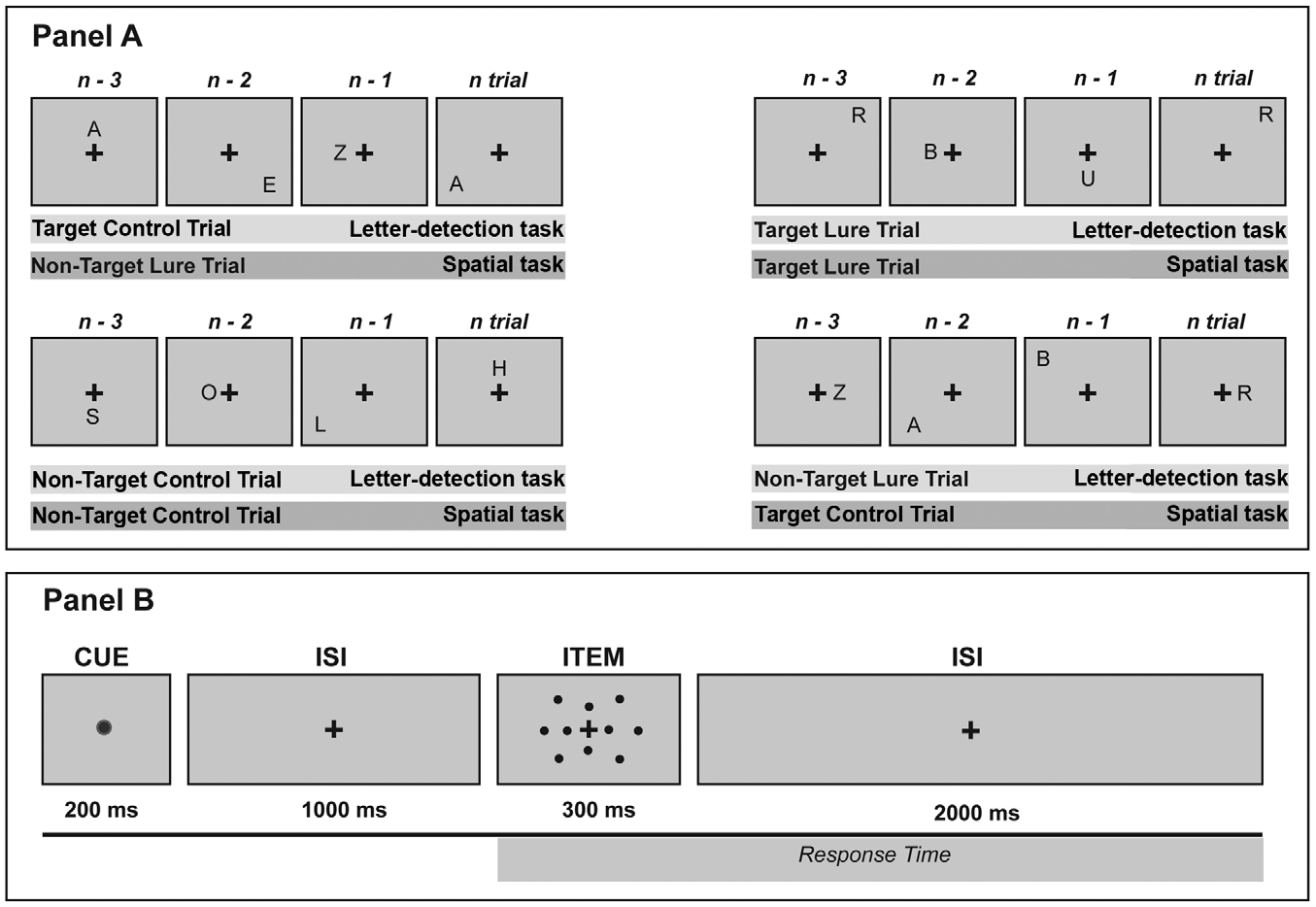
Experimental procedures. Panel A: Subjects were administered two versions (verbal and spatial) of a three-back memory task with identical stimuli (letters) presented at different locations of the screen. Four examples of the different kinds of trials used in the study are presented. Panel B: The time line of a single trial.

The same kind of manipulation was employed on some target trials (“Yes” responses). In both the letter-detection and spatial conditions, on some trials, the stimulus matched the three-back item on both identity and location features. We hypothesized that this could boost item familiarity and recollection, thus, resulting in a facilitation effect. We labeled these kinds of target trials as “target-lures”. All the other target trials, in which stimuli were matched only on the relevant domain, were identified as “target control” (Figure 1A).

Besides the WM task, participants were also assessed with the Raven's Advanced Progressive Matrices (RAPM; [26]). RAPM is a test of visuo-spatial abstract reasoning and it has been proven to load highly on the g factor. It is commonly considered a reliable measure of fluid reasoning (gf) – i.e. a factor belonging to the second broad stratum together with the crystallized intelligence as depicted in the hierarchic model of cognitive abilities [1].

### Experimental procedures

A PC-compatible computer running Cogent 2000 (developed by the Cogent 2000 team at the FIL and the ICN, University College London, UK) under Matlab (The Mathworks Company, Natick, MA, USA) controlled stimuli presentation and data acquisition. Items were chosen from a pool of 10 different uppercase letters. Notably, in order to minimize the number of stimuli features, we decided to use only uppercase letters instead of randomly changing between upper and lowercase. However, to ensure the use of verbal codifications of the stimuli, we explicitly asked participants to adopt such an encoding process. The location of the presented letter was selected among 10 different positions on the screen, which were spaced around the circumferences of two imaginary circles centered on the cross (see Figure 1B: ITEM, the dots represent the different locations). Each letter and stimulus position had the same probability of occurrence within each block (10%) and was pseudorandomly selected in order to accomplish experimental manipulations.

Participants were seated in a comfortable chair in front of a 15-inch computer screen at a distance of 50 cm. A computer mouse was used to acquire subjects' responses. Volunteers were instructed to respond with their right dominant hand by pressing the left button underneath their index finger, for a “target” trial (“Yes” response), and by pressing the right button under the middle finger, for a “no-target” (“No” response) stimulus. Before data acquisition, subjects underwent a training session that consisted of two 18 trials blocks, one for the spatial and one for the letter-detection condition. Training blocks were repeated if participants expressed or displayed difficulty in understanding or executing the instructions. After the training, all participants completed two experimental blocks for each of the WM domains. The order of the blocks was counterbalanced across WM domains and sex. Each of the experimental block consisted of 103 trials (3 starting stimuli followed by 100 test items) that were preceded by instructions relevant to the task domain (letter-detection or spatial). A single trial lasted for a total of 3.5 s and started with a warning stimulus (a yellow dot located at the center of the screen; “cue”). The cue was displayed for 200 ms and was followed by a white fixation cross positioned at the center of the screen. After 1 s the offset of the cue, an item (a letter in any of the ten position) appeared for 300 ms and was immediately followed by a white fixation cross lasting for 2 s (Figure 1B). Matches occurred in 36% of the trials, half of which were lures and the other half were controls. Non-match stimuli had a probability of occurrence of 64%. Among the non-matches trials, 20% were lures and the remaining 80% were controls (Figure 1A).

RAPM was administered individually, in a separate session. Specifically, subjects had to solve a total of 48 problems (12 for set I, 36 for set II) and didn't have any limit in time. Individual total raw scores (number of total correct responses, set I plus set II) were used for the analyses.

### Statistical analysis

In the first step of the analysis we tested whether information irrelevant to the goal of the task could bias the ongoing performance. We hypothesized: 1- the occurrence of two main effects: interference and facilitation; 2- that these phenomena should be reflected by variation of the individual performance between lures and controls at non-target and target trials, respectively. Specifically, on non-target trials in which the stimulus matched the three-back item on the irrelevant domain, we expected subjects to be slower in reaction time and less accurate as a consequence of high attentional control. On target-trials, in which stimuli were matched on both the relevant and irrelevant domains, we expected an enhancement in accuracy together with a decrease in mean reaction time. To test our hypotheses, we performed an analysis of variance (ANOVA) for repeated measures with a 2X2X2 factorial design where task (letter-detection and spatial), stimulus (target and non-target) and type (lures and controls) were the within subjects factors. Correct Responses (CR) and Reaction Time (RT) were entered as dependent variables in two separate ANOVA procedures. T-test was used for post-hoc comparison when needed.

In order to study differences in sensitivity among types of trial, we computed d′ indexes using the following formula [27]:
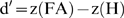
where z(FA) and z(H) are the z scores that correspond to the right-tail p-values represented by false alarm rate (FA) and hit rate (H). D′ indexes were computed for lure and control trials and for both the letter-detection and spatial tasks. D′ indexes were entered as dependent variables in 2X2 ANOVA for repeated measure where task (letter-detection and spatial) and type (lures and controls) were the within subjects factors.

A second step of the analysis sought to test the effects of task-set inertia on subjects' performance. All the subjects were exposed to both tasks in one single session. Thus, it might happen that the biasing effect due to the irrelevant information might be a reflection of previous exposure to a task in which that dimension was relevant. For instance, the effects coming from information about stimulus locations during the execution of the letter-detection task might be a consequence of a previous execution of spatial task and might reflect a failure in shifting completely attention to the new relevant dimension. Even if we controlled task-set inertia effects by counterbalancing task administration across domains and gender, we ran additional analyses on accuracy and reaction times after splitting the entire group (N = 60). We sorted subjects into two sub-samples (letter detection, N = 30; spatial, N = 30) on the basis of the type of task each participant first performed. We then computed percentage of accuracy and RTs only for the first block (either letter detection or spatial) and entered these variable into two separate 2X2X2 ANOVA where stimulus (non-target and target) and type (lure and control) were the within subjects factor, and group (letter detection and spatial) was the between subjects factor. Fisher's least significant difference (LSD) tests were used for post-hoc comparison when needed.

Similarly to the analysis performed on the entire group, we computed d′ indexes also for the two separate sub-samples and then tested the differences with a 2X2 ANOVA where type (lure and control) was the within subject factor and group (letter detection and spatial) was the between subjects factor.

A third step of the analysis sought to verify whether individuals' intelligence could predict the biasing effects. Independently for the spatial and letter-detection tasks, we first calculated correlations (Pearson index) among correct responses at the different types of trial (target controls, target lures, non-target controls, non-target lures) and RAPM. Then, to account for the common variance between interference control and general cognitive effort we computed partial correlation between non-target lure and RAMP by controlling for the effect of “non-target control”. Finally, those indices showing significant correlations with gf measure were entered into a stepwise regression procedure with the RAPM score as the dependent variables.

The relation (Pearson index) between individual differences in gf and sensitivity to the signal was also computed for lure and control trials and for the letter-detection and spatial tasks after eliminating those subjects having negative d′ values of d′ value near zero (<0.01).

## Results

### Information along the irrelevant domain can bias the ongoing performance

**Figure 2:**
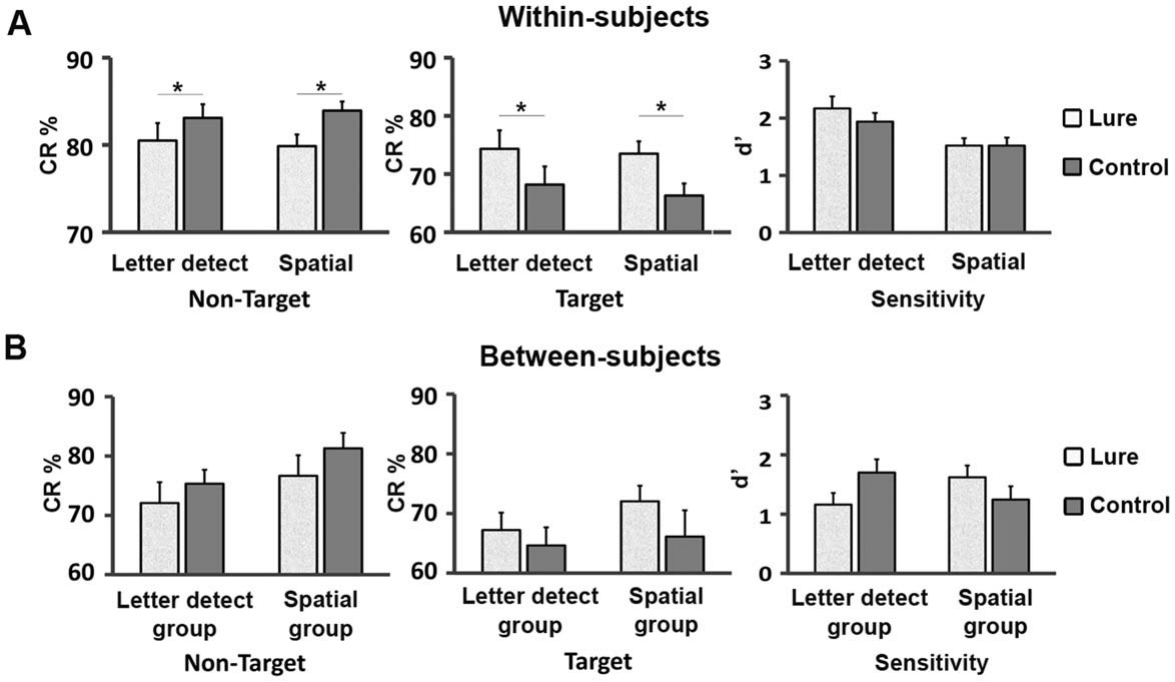
Interference and Facilitation effects. Results of the ANOVAs computed on accuracy (CR %: percentage of correct responses; d′: sensitivity index) at non-target and target trials for both the letter-detection and spatial three-back tasks. Panel A: Results of the analyses conducted on the entire group (within-subjects; N = 60); Bars represent the group-means for the lure and control trials; * Accuracy: p<.005; t-test. Panel B: Results of the analyses conducted on the two groups (between-subjects; each group N = 30); Bars represent the group-means for the lure and control trials.

Behavioral performance varied significantly between lure and controls trials in both the spatial and letter-detection WM tasks. The analysis conducted on the correct responses revealed a significant interaction among the three factors (taskXstimulusXtype, F(1,59) = 5.48, p = 0.023). The results are summarized in Figure 2A.

First, we demonstrated the occurrence of interference due to irrelevant information. Among the non-target trials, we found that lure trials were significantly more difficult than control trials. This was evident in both the letter-detection (t-test; t_(59)_ = 2.92, p = 0.005) and the spatial (t-test; t_(59)_ = 5.54, p<0.001) tasks, even if this latter was more challenging then the former, as revealed by the main effect we obtained for the factor task (F_(1,59)_ = 18.8, p<0.001). In other words, subjects showed more difficulties when the non-target stimulus (“No” responses) matched the three-back item on the irrelevant domain. Second, we found that irrelevant information had a facilitation effect on target trials. Participants showed an enhancement of accuracy at lures as compared to the control trials. This facilitation effect was evident in both letter-detection (t-test; t_(59)_ = −3.27, p = 0.003) and spatial (t-test; t_(59)_ = −3.99, p = 0.002) tasks. Subjects' accuracy was higher when the target stimulus (“Yes: response) matched the item seen three trials previously on both the relevant and irrelevant dimensions.

The results from the RT analysis were consistent with the findings reported so far. We revealed a significant interaction of stimulus and type factors (F_(1,59)_ = 9.20, p = 0.004). On non-target trials, the time to respond to lure stimuli was significantly higher than the RTs in the controls (univariate statistic for planned comparison, F_(1,59)_ = 4.2, p = 0.05). On the opposite, on target trials, subjects were faster when responding to a lure items as compared to control stimuli (univariate statistic for planned comparison, F_(1,59)_ = 5.29, p = 0.02). This last finding can be a reflection of redundancy target effect that is a decrease of reaction time when target detection is driven by simultaneous presentation of a stimulus. Indeed, the match between the current item and the three-back stimulus in terms of both their location and identity can be seen as a simultaneous presentation of a target along two separate dimensions [28], [29]. No main effect of the task factor (F_(1,59)_ = 1.09, p = 0.3) was found. Descriptive statistics (means and standard errors) for RTs are reported in Table 1.

**Table 1:** Descriptive statistics for the variables entered in the ANOVAs.

		Mean	S.D.	Skewness	S.E. Skew	Kurtosis	S.E. Kurt
**Within-subjects**	**RAPM**	36.27	6.48	-.61	.31	.86	.61
	**L-D non-target control**	83.03	12.13	-.65	.31	-.10	.61
	**L-D non-target lure**	80.54	15.45	-.73	.31	-.11	.61
	**L-D target control**	74.91	14.97	-.42	.31	-.80	.61
	**L-D target lure**	78.84	14.57	-.42	.31	-.99	.61
	**S non-target control**	80.91	14.00	-.80	.31	.29	.61
	**S non-target lure**	74.83	16.38	-.35	.31	-.79	.61
	**S target control**	66.30	18.86	-.89	.31	.59	.61
	**S target lure**	73.47	12.74	-.61	.31	-.06	.61
	**L-D d′ lure**	2.17	1.57	1.51	.31	2.86	.61
	**L-D d′ control**	1.94	1.20	1.34	.31	2.16	.61
	**S d′ lure**	1.52	.99	1.33	.31	2.76	.61
	**S d′ control**	1.52	1.09	.66	.31	1.51	.61

		Mean	S.D.	Skewness	S.E. Skew	Kurtosis	S.E. Kurt
**Between-subjects**	**L-D RAPM**	35.73	5.78	-.40	.43	.33	.83
	**L-D non-target control**	75.34	12.87	-.44	.43	-1.01	.83
	**L-D non-target lure**	72.08	19.28	-.74	.43	-.06	.83
	**L-D target control**	64.63	16.60	-.52	.43	-.09	.83
	**L-D target lure**	67.22	15.87	-.22	.43	-1.10	.83
	**L-D d′ lure**	1.25	1.10	1.08	.43	2.98	.83
	**L-D d′ control**	1.16	.71	-.29	.43	.41	.83
							
	**S RAPM**	36.80	7.17	-.82	.43	1.31	.83
	**S non-target control**	81.29	14.30	-.69	.43	-.21	.83
	**S non-target lure**	76.67	19.00	-.87	.43	.12	.83
	**S target control**	66.11	24.12	-1.07	.43	.39	.83
	**S target lure**	72.04	14.26	-.29	.43	-.39	.83
	**S d′ lure**	1.70	1.34	.99	.43	.56	.83
	**S d′ control**	1.62	1.35	.56	.43	.20	.83

L-D: Letter detection; S: Spatial.

The analysis conducted on d′ indexes revealed that sensitivity was higher in the letter-detection task as compared to the spatial one (main effect of task factor; F_(1,59)_ = 17.74; p<0.001). No significant differences were found between lure and control trials (main effect of type factor; F_(1,59)_ = 3.58; p = 0.063) even if a trend was evident, with sensitivity being higher at lure trials in the letter detection task (Figure 2A). No significant interaction between task and type factors (F_(1,59)_ = 2.36, p = 0.12) was found. Table 2 (upper part) reports the descriptive statistics for the variables used in the analyses described above.

**Table 2:** Descriptive statistics for the Reaction Times of the entire group (N = 60) and of the two separate groups (N = 30).

	WITHIN-SUBJECTS							
	Letter Detection task				Spatial task			
	non-target		target		non-target		target	
	lure	control	lure	control	lure	control	lure	control
**Mean**	967.384	964.374	929.490	964.633	974.196	950.933	910.762	918.270
**S.D.**	259.749	254.316	265.893	273.872	259.224	241.570	235.907	250.811

	BETWEEN-SUBJECTS							
	Letter Detection group				Spatial group			
	non-target		target		non-target		target	
	lure	control	lure	control	lure	control	lure	control
**Mean**	1082.333	1070.759	1067.100	1092.918	1003.397	955.150	899.015	954.985
**S.D.**	289.625	274.801	304.781	285.264	292.523	247.655	222.553	259.883

### Cross-domain interference and task-set inertia

The between-group analyses conducted on correct responses and RTs are in line with the findings reported so far. The analysis on accuracy did not shown any significant differences between the two groups (main effect of group factor; F_(1,58)_ = 1.53; p = 0.22), We found a main effect of the factor stimulus (F_(1,58)_ = 16.94; p<0.001) and a significant interaction between the factors stimulus and type (F_(1,58)_ = 7.14; p = 0.009). Similar to the results obtained for the entire group, we revealed that subjects were less accurate at non-target trials when the three-back item matched the current stimulus on the irrelevant domain (p = 0.06). On the other, hand hit rate was significantly higher for the target lures as compared to target controls (p = 0.05). The obtained findings are displayed in Figure 2B and the descriptive statistics are reported in Table 2 (upper part). Even if the difference between non-target lure and non-target control trials did not reach significant p value, the trend is clear and is in line with the original findings. In addition we have to take into account the small sample size (30 instead of 60) and the decreased number of trials (100 instead of 200) used to estimate subjects' means.

In regards to the RTs, we observed an interaction between the factors group and type (F_(1,58)_ = 4.62, p = 0.03). Indeed, we found that subjects were faster when the current stimulus matched the three-back item along the irrelevant dimension only when performing the spatial task (p = 0.009; see Table 1 for descriptive statistics).

The between-group analysis conducted on the d′ indexes did not show any significant differences (Figure 2B).

### Fluid reasoning predicts the ability to override interference coming from the irrelevant domain

The second aim of the current investigation was to verify whether individual differences in gf played a role in the modulation of these cross-domain biasing effects.

As shown in Table 3, accuracy at non-target trials (lure and control) for both the letter-detection and spatial tasks significantly correlated with gf. On the contrary, no significant correlations were found between RAPM scores and accuracy in target trials for the two WM tasks. In addition, no correlations were found between RAPM and reaction time (Verbal: “non-target control”, − 0.08; non-target lure, − 0.05; “target control”, − 0.04; target lure, − 0.04; Spatial: “non-target control”, − 0.14; non-target lure, − 0.18; “target control”, − 0.09; target lure, − 0.06;).

**Table 3:** Correlation analyses between Fluid intelligence and performance at the WM task.

	LETTER-DETECTION TASK			
	non-target		target	
	lure	control	lure	control
**RAPM** (zero-order)	**0.571** *	**0.490** *	0.162	0.222
**RAPM** (partial)	**0.348** ^	—	—	—

	SPATIAL TASK			
	non-target		target	
	lure	control	lure	control
**RAPM** (zero-order)	**0.534** *	**0.449** *	0.032	-0.177
**RAPM** (partial)	**0.324** ^	—	—	—

*p = .0001;

^p = .01.

Besides tapping interference, our paradigm also engages other executive processes that might account for the observed correlations, at least to a partial extent. The encoding and maintenance of new information, along with the updating of the items held in memory and their active manipulation, are some of these processes that might depend on gf level. In order to account for the effect of general cognitive effort, we computed correlation between RAPM and the accuracy at non-target lures by statistically controlling for correct response in “non-target control” trials. As reported in Table 3 we found significant correlation indices for both the letter-detection and spatial tasks suggesting an important role of individual level of gf in overriding interference after controlling for general cognitive effort.

**Figure 3:**
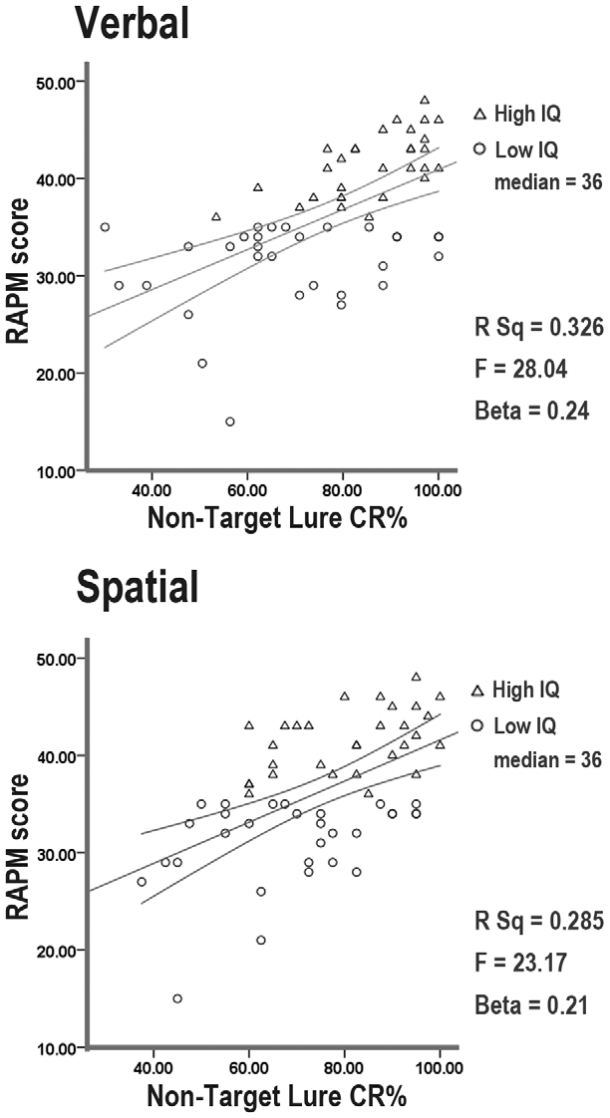
Gf and interference control. The two scatterplots between the Raven Progressive Matrices (RAPM) scores and accuracy (CR %: percentage of correct responses) at interference trials (non-target lure) are presented for both the verbal and spatial three-back tasks. Triangle and circle dots represent high-gf and low-gf subjects respectively (based upon the median of RAPM scores). Statistics of the regression analyses are also reported.

To confirm these findings, we ascertained which was the best predictor of RAPM among accuracy at lure and control trials. In order to avoid collinearity issues, we employed a stepwise regression model. For both the letter-detection and spatial tasks, the ability of overcoming interference (accuracy in non-target lure trials) was the best predictor of individual fluid intelligence level (Figure 3; p<.001).

Gf showed significant correlation with sensitivity at lure and control trials for both the letter-detection and spatial tasks (letter-detection lure r = 0.353, p = 0.006, N = 59; letter-detection control r = 0.414, p = 0.001, N = 59; spatial lure r = 0.399, p = 0.002, N = 59; spatial control r = 0.416, p = 0.002, N = 53).

## Discussion

In this study we investigated the relationship between fluid intelligence (gf) and a specific form of cognitive bias, i.e. the bias exerted by task-irrelevant feature of a salient stimulus. We revealed that information along a dimension that is irrelevant for task goals, and could therefore be ignored as per task instructions, can either enhance (facilitation) or disrupt (interference) subjects' performance. Furthermore, we showed that the ability to override interference effect across distinct domains depends on gf, thus suggesting a pivotal role of fluid intelligence in the active and efficient maintenance of stimuli representations along the task-relevant domain.

As recently pointed out [25] an implicit assumption underlying many previsions studies (see [25] for a review) was that irrelevant characteristics of a stimulus would be easily excluded from the ongoing processing. For instance, in WM paradigms like ours [11], [21], [30]–[33], one could reasonably assume that item locations would not be processed in the identity task, and that letter identity would not influence execution during the spatial task. This idea stems from the well-established cognitive theories that consider WM as partitioned into several domain-specific resources [17], as well as from studies showing a functional dissociation across brain regions of verbal and spatial WM components ([19]–[21] for a review of lesion studies: [22]). However, this view has been recently challenged by a number of investigations [15], [18], [23]–[25], [31] that provided evidence for domain-general mechanisms in WM functioning. In accordance with, and expanding these novel findings, we found that information coming from the irrelevant domain indeed significantly affects group performance, even when subjects are explicitly asked to focus on the relevant stimuli feature. More specifically, we observed two different and opposite effects, namely interference and facilitation, which are likely consequences of the trade off between assessment of familiarity and explicit recollection processes [13], [14]: on one hand, interference might be a reflection of the conflict between familiarity and recollection and the prioritizing of the former over the latter in case of incorrect responses; on the other hand, facilitation would reflect the synergic action between the two memory retrieval processes.

To our knowledge, this is the first work showing direct evidence of cross-domain biasing effects in a single-task paradigm. Cross-domain interference has been previously reported in the context of dual-task experiments in which subjects were asked to execute two distinct tests (for instance a verbal and a spatial task) in parallel. Interference was measured as a change in performance in the primary task as a function of the secondary one. It has been shown that a set of auditory-verbal items [34], [35] as well as a set of non-verbal acoustic stimuli [36] could actually disrupt the performance during a visuo-spatial task. Moreover, some authors have recently demonstrated that visual WM can be hampered by verbal stimuli even when the interfering task did not require covert responses [37]. Besides the dual-task paradigms, a previous investigation has reported cross-domain interference in two-back WM task similar to ours [38]. However, the conclusions were based upon indirect evidence of such an effect. This TMS study showed that, on average, group performance during sham stimulation was worst in terms of accuracy and reaction time when the presented stimuli varied along verbal and spatial domains as compared to the task in which the same stimuli varied only along one single dimension at the time (verbal or spatial). The analyses conducted on sensitivity indexes did not show any significant differences between control and lure trials. This finding is in line with the results obtained from the analyses on correct responses and might be a reflection of the opposite effects (facilitation and interference) that the irrelevant feature exerted on target and non-target trials. Sensitivity indexes reflect the difference between the hit and false alarm rates: in the current study whereas the irrelevant information increased the false alarm rate during the lure trials it also increased the hit rate at target lures thus making the difference between the false alarm and hit distributions similar to the difference between the two at control trials.

With regard to the trade off between familiarity assessment and explicit recollection, attentional control plays a fundamental role. Executive control is especially needed when subjects have to solve the mismatch between the two processes, in order to protect relevant memory contents and, eventually, to attain task-goals. Interestingly, our results showed that this ability is not similarly distributed across subjects but varies as a function of individual differences in fluid intelligence. We found that accuracy at non-target lure trials significantly correlated with gf, even after controlling for general cognitive effort that is: high gF individuals will show a reduced drop in performance on lure trials relative to controls, compared to lower gF individuals. In addition, no differences across subjects were found at target lure trials – that is facilitation trials- thus suggesting that gf has a limited role when familiarity and recollection coincided. Another possible explanation of the biasing effects observed here and of the relations among interference, facilitation and gf level is that irrelevant features of the three-back stimulus can provide useful information for a correct identification of the current item. Knowing whether the current item is a lure or a control can actually increases the chance of guessing the status along the relevant dimension (target or non-target). In fact, the chance that a lure stimulus is a target on the relevant dimension (18%) is higher then the chance of being a non-target (12.8%). Similarly, the probability that a control stimulus is a non-target along the relevant domain (51.2%) is higher then the chance of being a target (18%). In case the active retrieval of an item along the relevant domain might failure, knowing the status along the irrelevant dimension (lure vs. control) might increase the chance of guessing. Moreover, the positive correlations between gf and sensitivity indexes suggest that low gf individuals provide more false alarms and that they are more susceptible to this conditional probability [39].

However, besides the theoretical framework used for data interpretation (familiarity/recollection vs. conditional probability), what is clear is that the irrelevant features of the stimulus are actively processed, most likely because they provide useful information for the correct execution of the task.

The positive correlation between gf and interference control has been reported in previous studies using single-domain *n*-back WM task [9], [12], [15]. Furthermore, it has been shown that interference control positively correlated with WM capacity [40], [41], which is thought to capture similar skills as fluid intelligence [42]. Nonetheless, the current investigation is the first to point out the pivotal role of fluid intelligence in overcoming the deleterious effects interference across distinct domains. However, future studies are needed to investigate whether the gf variance explained by cross-domain bias overlap with the one accounted by interference within the same domain. A possible interpretation of our findings is that higher gf participants took advantage from the use of a strategy of interference anticipation and prevention that might be implemented through the enhancement of goal-related and task-relevant feature representation in WM. In fact, our task and the explicit instruction to focus on the relevant domain, might have favored the use of such a strategy. Although this conclusion in not based on direct evidence of such cognitive mechanisms, previous results support our hypothesis. It has been recently demonstrated that, when expected, interference in WM could be *proactively* controlled through the enhancement of memory representations of relevant information during the retention interval [9]. Moreover, the study above mentioned clearly showed that such ability was associated with higher gf level. Still, a different cognitive mechanism can account for our findings. Other authors revealed that proactive interference control could be achieved via active inhibition of task-irrelevant stimulus features [38]. However, if that were the case, along with the positive correlation between gf and interference, we would also expect, a negative correlation between fluid intelligence and facilitation, as consequence of the suppression caused by the inhibition processes of the synergy between familiarity and recollection. Our data did not show the presence of such a correlation. Nonetheless, further studies are needed to verify our hypotheses.
